# Congenital Urethral Fistula: A Case Report and Literature Review

**DOI:** 10.1155/2020/8862806

**Published:** 2020-09-30

**Authors:** Aamer Alghamlas, Frédéric Auber, Yann Chaussy

**Affiliations:** ^1^Department of Pediatric Surgery, CHU Besançon, F-25000 Besançon, France; ^2^Nanomédecine, Imagerie, Thérapeutique EA 4662, Université Bourgogne Franche-Comté, F-25000 Besançon, France

## Abstract

Male congenital urethral fistula is an extremely rare condition. It is characterized by an abnormal opening of the ventral aspect of the penis. We report the case of a 1-month-old boy with congenital urethral fistula. We will describe the surgical technique, postoperative results, and literature review.

## 1. Introduction

Male congenital urethral fistula is an extremely rare condition [1–3]. It is usually an isolated anomaly but sometimes associated with genitourinary or anorectal anomalies [1, 3]. It is characterized by fistulisation of the penile urethra to the skin with a normal urethral meatus [3]. Sometimes, it can be confused with hypospadias [4]. We present the case of a 1-month-old boy with congenital urethral fistula associated with a genitourinary anomaly (right cryptorchidism).

## 2. Case Presentation

**Figure 1:**
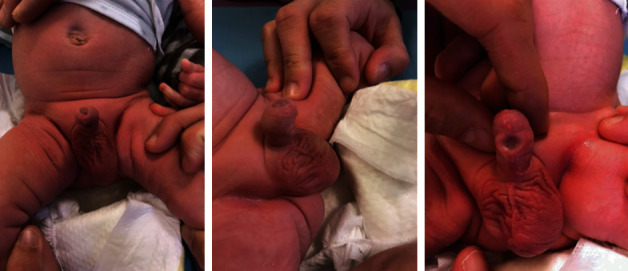
Clinical examination at the age of 1 month: an undamaged urethral wall with large corpus spongiosum and skin defect on the ventral aspect of the penis shaft. Associated right cryptorchidism.

A 1-month-old male was referred to our center for penile anomaly discovered during a routine neonatal examination. The twin pregnancy was unremarkable, and he was born at 38 weeks of gestation (birth weight 2350 g). He had no previous history of circumcision, surgery, or trauma. The clinical examination at 1 month of age found a normal urethral opening at the tip of the glans, normal foreskin, localized hypoplasia of the corpus spongiosum, and skin on the ventral penis surface. There was no ventral chordee. There was initially an undamaged urethra visible through the corpus spongiosum and skin defect on the ventral aspect of the penile shaft (Figure 1). We found also a right cryptorchidism with the testis in the inguinal area. There was no anorectal anomaly. The parents noticed a bulge on the ventral penis through the skin defect during urination although the patient urinated from the normal urethral meatus at the tip of the glans. We suspected the diagnosis of incomplete congenital urethral fistula because there was a local defect of corpus spongiosum and skin adjacent to a preserved urethral wall. The abdominal ultrasound was normal.

**Figure 2:**
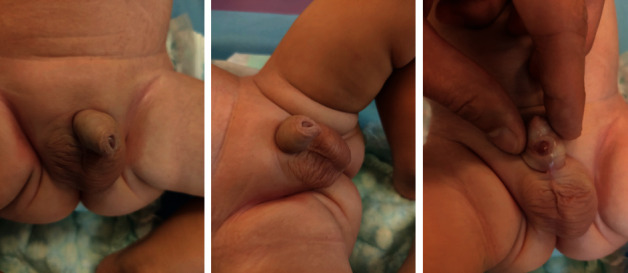
Clinical examination at the age of 3 months: urethrocutaneous fistula occurs after the rupture of the urethral wall. The patient is urinating from both the normal urinary meatus at the tip of the glans and the ventral urethrocutaneous fistula.

At 1 month and 1 week of age, the parents noticed a rupture of the urethral wall and urination from both the normal urethral opening and the urethrocutaneous fistula. The clinical examination found an oval-shaped opening measuring approximately 1.5 cm in length and 0.5 cm in width (Figure 2).

**Figure 3:**
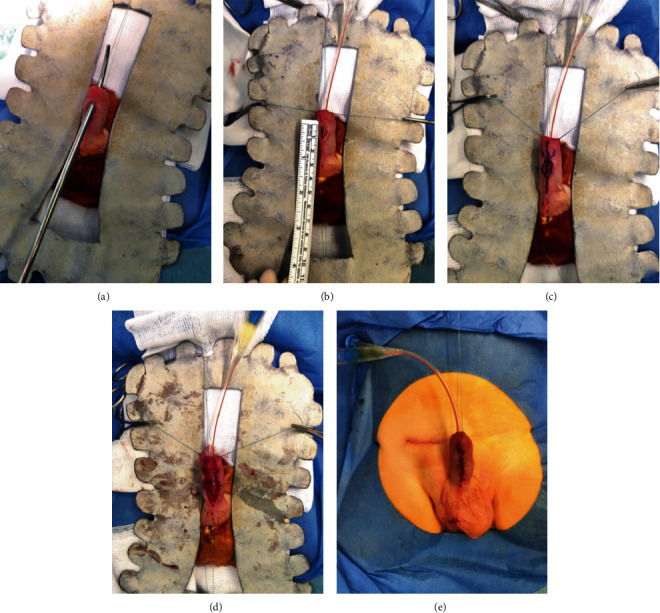
Surgical correction at the age of 6 months. Permeability of the distal urethra is tested with a urethral dilator until 12 Fr (a). An 8 Fr Foley catheter is inserted, and the congenital urethral fistula is measured (b). Drawing of skin incision (c). Primary fistula closure is performed by the tubularization technique, and spongioplasty is done to cover the urethroplasty (d). Final postoperative result (e).

Surgical correction was performed at the age of 6 months. After correction of the right cryptorchidism, testing of the distal urethra with a urethral dilator until 12 Fr demonstrated the absence of stricture; then, an 8 Fr Foley catheter was passed into the bladder. A longitudinal ventral incision circumscribing the fistula was performed. The penile shaft skin was ventrally degloved. We noticed a normal proximal and distal urethra surrounded by the corpus spongiosum. There was a large defect in the corpus spongiosum. The urethral plate was well developed. The urethroplasty was performed using a tubularization technique with a continuous 7.0 polydioxanone resorbable suture. The corpus spongiosum was then dissected in order to perform spongioplasty, covering the urethroplasty. The subcutaneous tissue was then used as an additional layer to cover sutures to limit the risk of fistula recurrence. Finally, the skin was longitudinally closed (Figure 3). The patient was discharged at day 2, and the Foley catheter was removed at day 6 on an outpatient basis.

**Figure 4:**
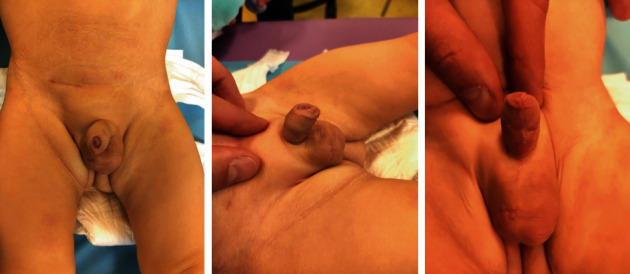
Postoperative results at 6 months: wound healing is complete without chordee and with a good esthetic outcome. The patient has normal voiding by urinary meatus without fistula recurrence.

Six months later, the patient had normal voiding without fistula recurrence. The parents had observed a normal stream via the urethral meatus without any leakage. The wound was completely healed without chordee and with good esthetic results (Figure 4). The right testis was located in the normal intrascrotal position.

## 3. Discussion

Congenital urethral fistula is a rare condition where an urethrocutaneous fistula occurs in the ventral aspect of the penis shaft with a normal urethral meatus. There are few reports of this congenital anomaly in literature [3]. The etiology and pathogenesis are uncertain, and several theories have been proposed. The male genitalia develop in a proximal-to-distal manner. As the penis forms from the elongation and enlargement of the phallus, the lateral walls of the urethral groove form from the ventrally located genital folds, which then fuse at the midline [5]. For Olbourne, fistulae located in the penile shaft probably reflect a focal or temporary defect in the urethral plate function. This would result in a complete defect or a partial deficiency of urethral fold fusion [2]. Another theory suggests that the misalignment of the glanular and penile urethra may explain the coronal type of congenital urethral fistula [1]. In addition, the anterior urethral fistula may be secondary to a blowout phenomenon of a urethral diverticulum. Consequently, this condition may lead to a defect in the corpus spongiosum or incomplete migration of the lateral spongy tissue of the inner genital folds with later erosion of the overlying skin [1, 6]. Our case supports this last hypothesis: the urethral wall was initially undamaged with a urethra bulging through the corpus spongiosum and skin defect during micturition. The pressure caused by the urinary stream led to the rupture of the thin urethral wall (which is not covered by corpus spongiosum) and subsequent urethrocutaneous fistula.

Congenital urethral fistula is usually an isolated disorder (with normal foreskin, normal urinary meatus, and no chordee). However, it can be associated with genitourinary anomalies (hypospadias-like characteristics, undescended testis, bifid scrotum, duplicated urethra, megalourethra, and penoscrotal transposition) or anorectal anomalies [1, 3]. Congenital anterior urethrocutaneous fistula is uncommon compared to posterior urethrocutaneous fistula which usually presents as a Y-type duplication of the urethra with anorectal atretic malformations [1]. It is important to differentiate between isolated congenital urethral fistula and urethral fistula with associated anomalies (particularly hypospadias-like anomalies or anorectal malformation) as the surgical treatment will be different.

The surgical approach to repair congenital anterior urethrocutaneous fistula depends on the type and size of a fistula. Primary fistula closure is considered if it appears that the distal urethra and spongiosum are normally formed, as in our case. Thus, surgical correction requires circumscription of the fistula and closure in multiple layers, similar to a urethrocutaneous fistula after hypospadias repair [1, 5, 7]. Various techniques, depending on the size and location of the fistula, have been proposed to perform urethroplasties such as Thiersch-Duplay urethroplasty, turned-down flap urethroplasty, and pedicled island tube or onlay urethroplasty [3, 4]. When the congenital urethral fistula is associated with deficient distal urethra or spongiosum, chordee, or hypospadias, formal hypospadias repair is recommended [1, 7]. The functional and esthetic results are usually good with a low risk of fistula recurrence when the surgical technique is correctly chosen [1].

## 4. Conclusion

Congenital urethral fistula is a very rare condition. Careful diagnosis and exploration for associated anomalies are necessary for good management. The surgical approach depends on the type and size of a fistula, with usually very good functional and esthetic results.

## Data Availability

The data used to support the findings of this study are included within the article.
